# Generation of a miniature pig disease model for human Laron syndrome

**DOI:** 10.1038/srep15603

**Published:** 2015-10-29

**Authors:** Dan Cui, Fang Li, Qiuyan Li, Jia Li, Yaofeng Zhao, Xiaoxiang Hu, Ran Zhang, Ning Li

**Affiliations:** 1 State Key Laboratory for Agrobiotechnology, China Agricultural University, Beijing 100193, China; 2 State Key Laboratory of Cellular Stress Biology, School of Life Sciences, Xiamen University, Xiamen, Fujian 361102, China

## Abstract

Laron syndrome is a rare disease caused by mutations of the growth hormone receptor (*GHR*), inheriting in an autosomal manner. To better understand the pathogenesis and to develop therapeutics, we generated a miniature pig model for this disease by employing ZFNs to knock out *GHR* gene. Three types of F0 heterozygous pigs (GHR^+/4bp^, GHR^+/2bp^, GHR^+/3bp^) were obtained and in which no significant phenotypes of Laron syndrome were observed. Prior to breed heterozygous pigs to homozygosity (GHR^4bp/4bp^), pig *GHR* transcript with the 4 bp insert was evaluated *in vitro* and was found to localize to the cytoplasm rather than the membrane. Moreover, this mutated transcript lost most of its signal transduction capability, although it could bind bGH. GHR^4bp/4bp^ pigs showed a small body size and reduced body weight. Biochemically, these pigs exhibited significantly elevated levels of GH and decreased levels of IGF-I. These results resemble the phenotype observed in Laron patients, suggesting that these pigs could serve as an ideal model for Laron syndrome to bridge the gaps between mouse model and human.

Laron syndrome is an autosomal disease characterized by dwarfism, frontal bossing, a small midface, moderate obesity and small genitalia^1^. Laron patients have extremely high levels of circulating growth hormone (GH), and very low levels of insulin-like growth factor I (IGF-I), and they exhibit no response to the administration of GH^2^,^3^. It has been confirmed that the gene defects for this syndrome lie in growth hormone receptor (*GHR*), and a variety of *GHR* defects have been subsequently identified^4^. Human GHR is a 638 amino acids transmembrane protein with an extracellular domain (mainly encoded by exon 3 through exon 7), a transmembrane domain (mainly encoded by exon 8) and an intracellular domain (mainly encoded by exons 9 and 10), which belongs to the cytokine receptor family^5^,^6^. The soluble form of GHR is GH-binding protein (GHBP) that corresponds to the extracellular part of it has been identified in plasma^7^, which is usually absent in most Laron patients although some patients with high or normal levels GHBP have also been reported.

The binding of GHR to GH initiates the GH-GHR signal pathway, resulting in the production of IGF-I and promoting the growth, development and immunity function of the organism. Upon binding GH, a conformation change in the receptor leads to the activation of the Janus kinase 2 tyrosine kinase and of GHR itself through phosphorylation, in turn stimulating the STATs, PI3-AKT and MAPKs signaling pathways^8^,^9^,^10^,^11^. Specifically, phosphorylated STAT5 can translocate to the nucleus and bind the promoters of IGF-I to facilitate gene expression. IGF-I produced in liver can bind to IGF-binding protein-3 (IGFBP3) and acid labile subunit (ALS) to form a ternary complex in blood, which can be transported to target tissues such as muscle, bone and adipose^12^. Thus, mutation of the *GHR* gene can exert a devastating influence on the growth and development of the body.

Defects in the *GHR* gene, including nonsense mutations, splice site mutations, frame shifts, deletions and missense mutations^13^, can impair the GHR signaling pathway, thereby resulting in the dwarfism seen in Laron syndrome. To date, the only therapeutic treatment for this disease is recombinant IGF-I, which has been shown to have various side effects, including hypoglycemia, thymic hypertrophy, snoring and hypoacusis^14^. To investigate the mechanism underlying this disease and to develop therapeutics for it, investigators have employed animal models such as the dwarf chicken and the Laron mouse^15^,^16^. However, the anatomical and evolutionary differences between humans and chickens limit the chicken’s utility as a model for Laron syndrome. Although the Laron mouse recapitulates many phenotypes of human Laron syndrome, important differences have been observed. For example, the fat distribution in females and males and the change in circulating glucose levels observed in Laron patients are not consistent with those in the Laron mouse^17^,^18^. In addition, bone growth is different between humans and rodents; sexual maturity induces epiphyseal closure to end the normal growth of longitudinal bone in human^19^, whereas longitudinal growth continues after sexual maturity in rodents.

To address the above question, we chose to create a pig model because of the pig’s widespread use in biomedical research^20^,^21^,^22^,^23^,^24^. Its large body size, large number of offspring, biological and physical similarities to human and relatively long life span render pig an ideal model for the study of Laron syndrome. For example, the longevity of pigs made it possible to investigate the pathogenesis of the disease and to assess the efficacy and side effects of the treatments. Additionally, the emergence of gene-editing tools such as ZFNs, TALENs and CRISPR/Cas9 eliminates the limits of generating genetically modified pigs using the low efficient homologous recombination method by labor-intensive screening^25^,^26^.

Here we used ZFNs to produce a human Laron syndrome disease model in miniature pigs by knocking out the *GHR* gene. The homozygous KO pigs resemble the phenotypes observed in Laron patients.

## Results

### ZFNs were efficiently employed to disrupt the pig *GHR* gene in fibroblast and obtain of F0 heterozygous pigs by SCNT

**Figure 1:**
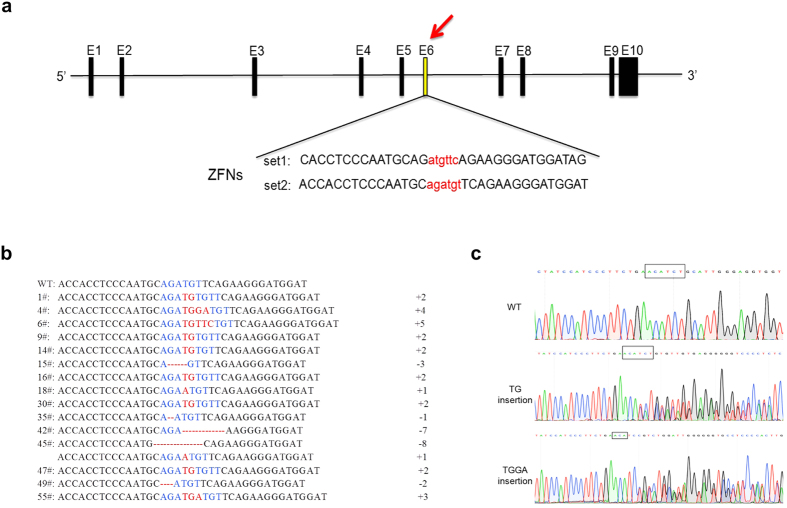
Obtaining F0 heterozygous pigs through SCNT by knocking out *GHR* gene with ZFNs. (**a**) Schematic representation of ZFNs targeting exon 6 of the pig *GHR* gene. E1–E10 represent the ten exons of the pig *GHR* gene, the lower-case characters and the upper-case characters are the spacers and the DNA binding sequences, respectively. (**b**) Identification of the cell clones for SCNT. The mutation types of *GHR* in different cell clones were small indels. (**c**) Identification of F0 heterozygous pigs by Sanger sequencing of PCR products. The results of 2 bp (TG) and 4 bp (TGGA) inserts are double peaks; WT indicates the result obtained from the wild-type pigs.

To investigate the activity of ZFNs in pigs, we designed two ZFNs for exon 6 of the *GHR* gene (Fig. 1a). We transfected ZFNs and the efficiency of disrupting the *GHR* gene was calculated by TA cloning and Sanger sequencing. Our results demonstrated that ZFNs set 2 had a higher activity than set 1 in pig fibroblasts for a range of ZFNs transfection amounts (Table 1), with the highest efficiency of 22.5% when the transfection amount reached to 4 μg. Moreover, there were no differences in ZFNs activity between different types of pig fibroblasts, and the optimal amount of ZFNs used in our study ranged from 4 μg to 6 μg (Table 2). Many potential off-target sites have been avoided by employing bioinformatics analysis in the experimental design, we transfected the fibroblasts from several species with ZFNs set 2 to determine the specificity of ZFNs targeting for the pig *GHR* gene. Blast results showed that 1–5 mismatches were found in cow, sheep, rabbit and human compared with the target sites on pig *GHR* (Table 3). No mutations were detected by Sanger sequencing with four or five mismatches, whereas a 3.4% mutation efficiency was observed with one mismatch for the target sites in rabbit (Table 3). The results were consistent with those obtained in cell clones used for SCNT in our work. We identified nine potential off-target sites by bioinformatics analysis, specifying a maximum of six mismatches at target sites in the pig genome, and no mutations were observed at the predicted off-target sites by T7E1 cleavage assay (data not shown).

Genetically modified pigs were produced by somatic cell nuclear transfer (SCNT). Miniature fibroblasts were co-transfected with vectors of ZFNs set 2 and the Neo^r^ gene, and then were selected with G418. Of the 45 G418-resistant colonies obtained, 15 were disrupted by ZFNs and colony 45# contained two disrupted *GHR* alleles (Fig. 1b). Colonies of 4# (4 bp insert), 1# (2 bp insert), 55# (3 bp insert) and 45# that had better quality and viability were chosen for SCNT. A total of 4377 reconstructed embryos were transferred into 11 surrogate pig recipients, of which only five surrogate pigs became pregnant. Fourteen piglets were born by the surrogate pig that received embryos from 1# colony, four piglets were born from 4# colony, two piglets were born from 55# colony, and no piglets were obtained from 45# colony. The genotypes of piglets in the F0 generation were determined by TA cloning and Sanger sequencing (Fig. 1c). We compared *GHR* mRNA expression among F0 pigs, and no significant difference was found between pigs with one allele disrupted and the wild-type controls using RT-PCR and qRT-PCR (Fig. S1a, 1b). There was no significant difference in body weight between *GHR* one allele disrupted pigs and the wild-type pigs (Fig. S1c). Neither GH was significantly increased nor was IGF-I significantly decreased in pigs with one *GHR* allele disrupted compared with the wild-type pigs (Fig. S1d, 1e).

### Validation of the function of the pig *GHR* transcript with a 4 bp insert *in vitro*

**Figure 2:**
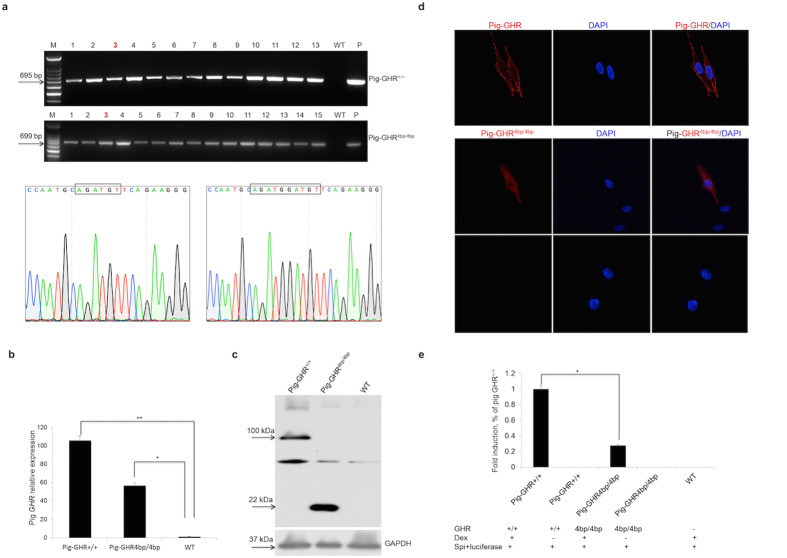
Evaluation of the pig *GHR* transcript with a 4 bp insert *in vitro*. (**a**) RT-PCR of the 4 bp insert and the wild-type pig *GHR* transcripts in stably integrated DF1 cell clones. Pig-GHR^+/+^ and Pig-GHR^4bp/4bp^ represent the wild-type and the 4 bp insert pig *GHR*, respectively; numbers represent the different cell clones obtained; WT represents the results obtained from un-transfected DF1 cells; P represents the plasmid control; and the sequencing results of the clones are also shown. (**b**) qRT-PCR of pig *GHR* mRNA expression in stably integrated DF1 cells. The expression levels were determined by the expression relative to *GAPDH* (an internal control). The data were combined from three independent experiments; the bars represent the means ± SD (n = 4–6 cell clones per group); **P < 0.01, *P < 0.05. (**c**) Western blot of pig GHR expression in stably integrated DF1 cells. The wild-type and mutated GHR are 100 kDa and 22 kDa, respectively; GAPDH was used as an internal control. (**d**) Determination of the localization of pig GHR in DF1 cells. Stably transfected DF1 cells were stained with the anti-Flag tag primary antibody (1:500) and Cy3-conjugated goat anti-mouse IgG antibody. DAPI (blue) staining indicates the nucleus. The data are representative of at least three independent experiments. Images were obtained with a 100x oil objective lens. (**e**) Dual-luciferase assay of the mutated pig GHR and the wild-type GHR. Dex represents Dexamethasone (200 nM); Spi+luciferase is the reporter vector used in this assay; GH (50 nM) was used to induce the signal transduction; the bars represent the means ± SD; *P < 0.05.

The pigs obtained with one disrupted *GHR* allele did not exhibit remarkable Laron syndrome phenotypes. However, the 4 bp insert in exon 6 of pig *GHR* will lead to a frame shift and thereby truncate the GHR protein, we therefore hypothesized that double alleles with the 4 bp insert could disrupt *GHR* gene in pigs. To get the *GHR* double alleles disrupted pigs by breeding of GHR^+/4bp^ pigs (defined as +/− pigs), we first validated the function of the *GHR* transcript with a 4 bp insert *in vitro*. Both the 4 bp insert pig *GHR* transcript (defined as Pig-GHR^4bp/4bp^) and the wild-type pig *GHR* transcript (defined as Pig-GHR^+/+^) were cloned from the liver of GHR^+/4bp^ pig, and then cloned into Psp72-AvITIT-c-flag vector with a Flag tag attached to the N-terminus of the fusion protein. DF1 cell clones with stable expression of the mutated *GHR* transcript or the wild-type *GHR* transcript were obtained, as confirmed by RT-PCR and sequencing (Fig. 2a). The mRNA expression of both the mutated and the wild-type pig *GHR* in DF1 cell clones were significantly higher than in un-transfected cells as determined by qRT-PCR (Fig. 2b). Using the Flag tag-specific primary antibody, we identified a 100 kDa and a 22 kDa protein corresponding to wild-type pig GHR and the mutated pig GHR, respectively (Fig. 2c). The stable integrated cell clones were also examined by immunofluorescence; the wild-type pig GHR was observed at the membrane in DF1 cells, whereas the mutated pig GHR localized to the cytoplasm instead of the membrane. This indicated that the mutated pig GHR altered the localization in the living cells (Fig. 2d).

To investigate the function of mutated pig *GHR*, a binding assay with bovine GH was performed *in vitro*. Both the mutated pig GHR and the wild-type GHR were able to bind to bovine GH, whereas the KD (1.3 × 10^−8^) of mutated pig GHR was reduced to approximately 1/5 of the KD (6.5 × 10^−8^) obtained for the wild-type pig GHR. To further evaluate the function of signal transduction capability of the mutated GHR, a dual-luciferase assay was conducted. We transfected DF1 cells that exhibited stable expression of mutated or wild-type pig GHR with the Spi-luciferase reporter. 50 nM human GH and 200 nM Dex were added to the culture media to induce the GH-GHR signal transduction. As shown in Fig. 2e, luciferase activity in cell lines expressing the mutated pig GHR was reduced four-fold compared with that in the cell lines expressing wild-type pig GHR. These results demonstrated that mutated pig GHR lost most of its signal transduction function *in vitro*. Therefore, we speculated boldly that GHR^4bp/4bp^ pigs (defined as −/− pigs) would lose most of the function of GHR and might mimic the phenotype observed in Laron patients.

### Homozygous mutant pigs obtained through breeding

**Figure 3:**
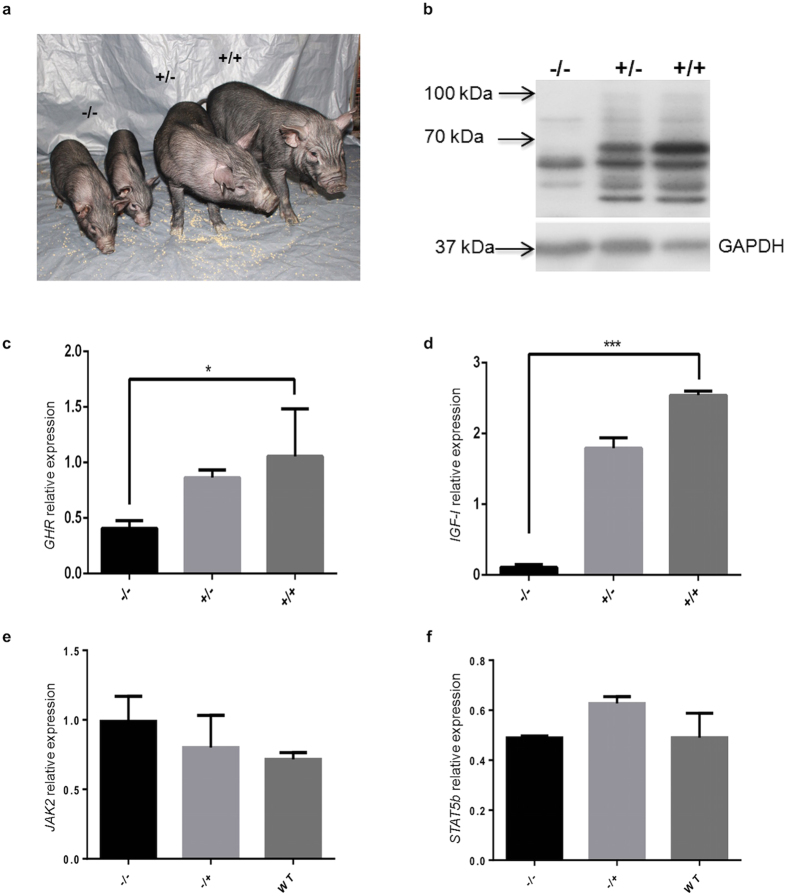
Knockout of the pig *GHR* gene in F2 homozygous pigs (GHR^4bp/4bp^ refers to −/−). (**a**) The −/− pigs have a smaller body size. (**b**) Western blot of GHR in F2 pigs. The −/−, +/− (GHR^+/4bp^) and +/+ (GHR^+/+^) represent different genotypes in the F2 generation. The expected sizes for pig GHR are 100 kDa (glycosylated) or 70 kDa (un-glycosylated); GAPDH was used as an internal control. The mRNA expression of *GHR* (**c**), *IGF-I* (**d**), *JAK2* (**e**) and *STAT5b* (**f**) genes in the F2 generation was determined by qRT-PCR. The expression levels were determined by normalizing the expression relative to *GAPDH*. The data were combined from three independent experiments; the bars represent the means ± SD (at least three pigs per group); ***P < 0.001, *P < 0.05.

We obtained F1 GHR^+/4bp^ pigs by breeding and the genotype was confirmed by T7E1 assay (Fig. S2), and we then obtained the −/− pigs by breeding of the GHR^+/4bp^ pigs of the F1 generation (Fig. 3a). *GHR* mRNA expression was significantly reduced in the −/− pigs (*P* < 0.05), while there was no significant difference between the +/− and +/+ pigs (refer to GHR^+/+^ pigs) (Fig. 3c, S3). Interestingly, expression of *IGF-I* mRNA was also significantly lower in the −/− pigs than in the +/− and +/+ pigs (*P *< 0.01) (Fig. 3d). However, there were no significant differences in terms of the expression of *JAK2* and *STAT5b* among the −/−, +/−, and +/+ pigs (Fig. 3e,f). The GHR protein was absent in −/− pigs (Fig. 3b). The 100 kDa protein observed was probably the glycosylated form of GHR, and the 70 kDa protein was un-glycosylated GHR. The extracellular domain of GHR (GHBP) was also absent in plasma of −/− pigs and no significant differences were observed between +/−, and +/+ pigs (Fig. S4).

### The −/− pigs exhibited reduced growth and similar biochemical features to those observed in Laron patients

**Figure 4:**
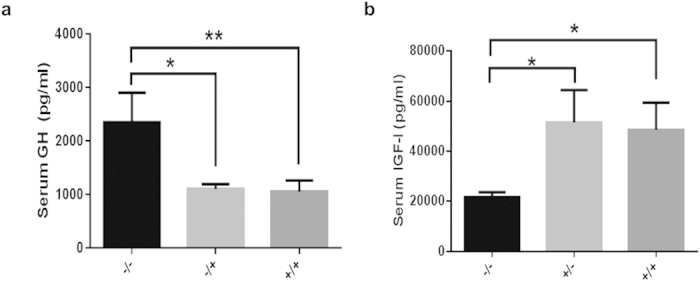
Growth retardation in the −/− pigs. Determination of body weight (**a**), body length (**b**), chest circumference (**c**) and body height (d in the F2 generations suggests growth failure in the −/− pigs. The −/−, +/− (GHR^+/4bp^) and +/+ (GHR^+/+^) represent different genotypes in the F2 generation; the bars represent the means ± SD (at least three pigs per group); *P < 0.05.

Before weaning, there were no significant differences in the weight and body size among the −/−, +/− and +/+ pigs. After weaning, the body size and weight of the −/− pigs started to be significantly smaller than either the +/− or the +/+ pigs and there was no significant difference between the +/− and +/+ pigs (*P *< 0.01). The weight pattern in the −/−, +/− and +/+ pigs was tracked over age progression (Fig. 4a). Moreover, body length was also significantly shorter in the −/− pigs compared with that observed for the +/− and the +/+ pigs (*P *< 0.01). No significant differences in body length were observed between the +/− and the +/+ pigs (Fig. 4b). Additionally, the body height and the chest circumference showed similar trends to body weight and the body length, which were significantly reduced in the −/− pigs compared with the +/− and the +/+ pigs (*P *< 0.01), while no significant difference between the +/− and the +/+ pigs was observed (Fig. 4c,d). The reductions in body size and body length could be attributed to the reduced growth of bones in the −/− pigs (Fig. S5). The size and weight of organs were also reduced in the −/− pigs (data not shown).

**Figure 5:**
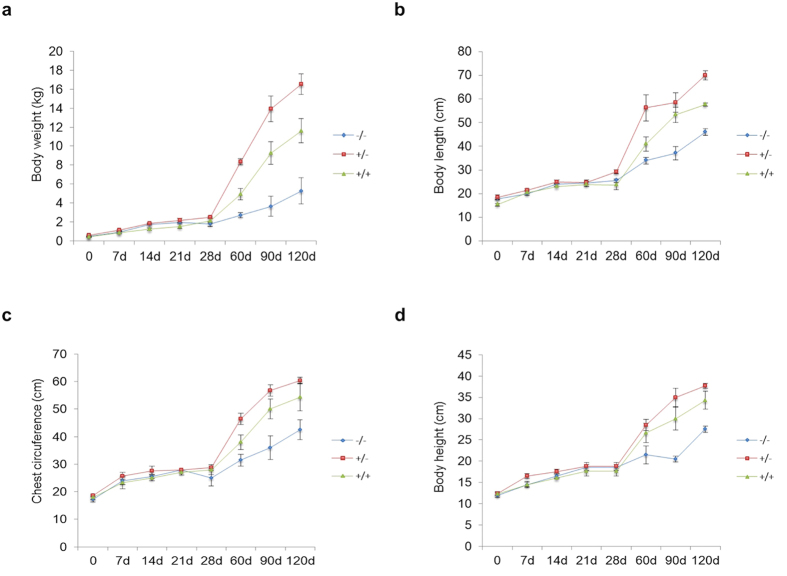
Biochemical changes in the GH-IGF-I axis. The −/− pigs showed significantly elevated level of GH (**a**) and reduced level of IGF-I (**b**). The −/−, +/− (GHR^+/4bp^) and +/+ (GHR^+/+^) represent different genotypes in the F2 generation; the bars represent the means ± SD (at least three pigs per group); **P < 0.01, *P < 0.05.

**Figure 6:**
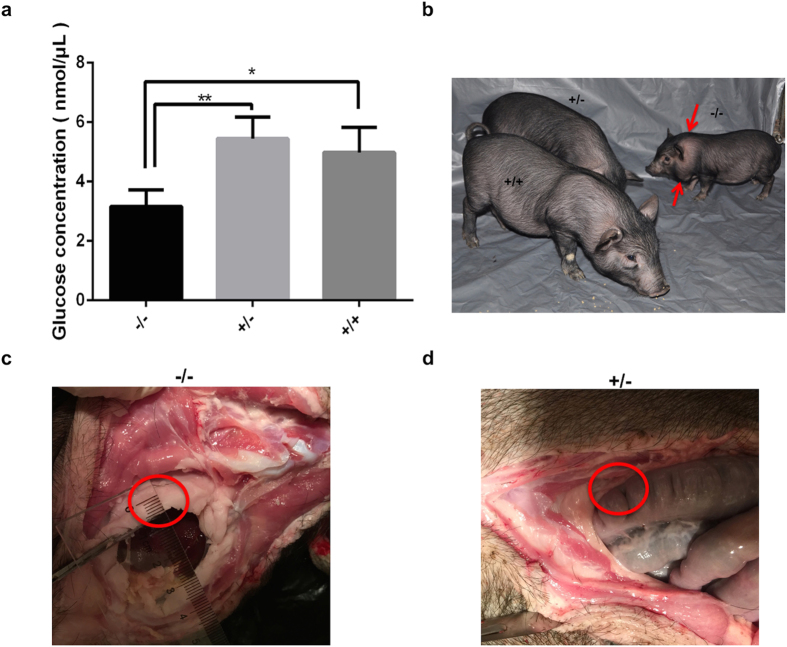
Glucose levels and obesity of the *GHR* homozygous KO pigs. (**a**) The levels of glucose were determined by a Glucose Assay Kit (ab65333, Abcam). The −/−, +/− (GHR^+/4bp^) and +/+ (GHR^+/+^) represent different genotypes in the F2 generation, the bars represent the means ± SD (at least three pigs per group); **P < 0.01, *P < 0.05. (**b**) The −/−, +/− (GHR^+/4bp^) and +/+ (GHR^+/+^) pigs at age of two months old, red arrows showed the obesity observed in homozygous KO pigs. The thick layer of abdominal fat (as shown by red circles) was observed in −/− pig (**c**), while no such fat layer was observed in +/− (GHR^+/4bp^) pig (**d**). The pigs in (**c**) and (**d**) were three-and-half-month-old.

GH in the serum of the −/− pigs was significantly increased (*P *< 0.05), whereas the GH concentrations in the +/− and the +/+ pigs did not differ significantly (Fig. 5a). In contrast to GH, serum IGF-I was significantly lower in the −/− pigs than that in the +/− or the +/+ pigs (*P *< 0.05), whereas IGF-I levels were comparable between the +/− and +/+ pigs (Fig. 5b). These results are consistent with those obtained in Laron patients. Significantly low levels of glucose were also observed in the −/− pigs (Fig. 6a), which was consistent with the hypoglycemia observed in Laron patients. We observed obesity in the *GHR* homozygous KO pigs of two months old as shown in Fig. 6b. Thick layer of abdominal fat in the three-and-half-month-old −/− pig (Fig. 6c) was also observed, whereas there was no such thick layer of abdominal fat in the +/− (Fig. 6d) and +/+ pigs. These results indicate the abnormality of glucose and lipid metabolism in −/− pigs.

## Discussion

Pig is an ideal biomedical models because of its biochemical and physiological similarities to humans as well as its larger body size and ease of handling, which will bridge the gaps between mouse models and human patients with its increasing utilizations in research. We chose the miniature pigs in this study for the following reasons. First, pigs posses many similarities with humans in the GH-IGF-I axis^27^. Second, in terms of growth, during embryonic development, key events such as the formation of the blastula and the appearance of the upper limb bud occur at the same time in the gestation of pigs and humans^28^; and regarding the postnatal growth, pigs share a similar growth curve to humans. Third, the similarities in the features of bone growth^29^,^30^, gastrointestinal tracts and digestive physiologies^31^,^32^ and the metabolism of glucose, lipids and proteins^33^ between pigs and human, allowing it a better model for studying the effects of GHR defects on bone growth and metabolism observed in Laron syndrome. Fourth, pigs will provide a better model for assessing the efficacy of treatments of Laron syndrome, since they have a high degree of similarities of skin and subcutaneous tissues to humans^34^, which could result in comparable pharmacokinetics in these species. We currently are conducting the breeding experiment to get enough homozygous KO pigs to perform the above-mentioned functional studies.

Researchers have identified more than 70 mutations among all ten exons of the *GHR* genes in Laron syndrome patients worldwide^35^. Both homozygous and compound heterozygous mutations of *GHR* can lead to growth failure, and compound heterozygous mutations usually cause dwarfism via a dominant negative effect. In our study, we chose to knock out the pig *GHR* gene at exon 6 to generate a Laron syndrome disease model mainly because exon 6 encodes part of the extracellular domain of GHR, thereby this deletion would abrogate the function of GHR by causing a frame shift. Another reason for this choice is that ZFNs can efficiently knock out genes by NHEJ at upstream exons.

Mature GHR protein contains 620 amino acids composing of an extracellular domain, a transmembrane domain and an intracellular domain. The extracellular domain contains seven cysteine residues and five potential N-linked glycosylation sites, while only three of these sites will glycosylate in pigs. The anticipated molecular weight of pig GHR is 70 kDa and glycosylated GHR will be 100 kDa after glycosylation of the three potential glycosylated sites. In our *in vitro* experiment, we observed a 100 kDa band for wild-type pig GHR, indicating the glycosylation of pig GHR in DF1 cells. The 4 bp insert in the *GHR* gene stops translation at site 195, suggesting that the mutated GHR only contains part of the extracellular domain and lost all of the transmemebrane domain as well as the intracellular domain. This could explain why the mutated protein was located in the cytoplasm rather than at the membrane in the immunofluorescence assay in our study. However, mutated GHR was still able to bind bovine GH though it contains only part of the extracellular domain. This binding can probably be attributed to the critical residues (W104, W169, I103, I105, I106, I165, R43, E44, D126, E127 and D164) for GH binding still maintained in the mutated GHR^36^.

STAT5, phosphorylated by GH signaling, is the transcription factor that could bind the Spi 2.1 promoter to facilitate the expression of the Spi gene in rat^37^. Our results suggested that the absence of the transmembrane and intracellular domain abrogated most of GH-STAT5 signal transduction. These results corroborate other impairments of STAT5 activation in GH signaling observed following truncation of the GHR intracellular domain^38^,^39^. The shortage of anchor sites (C-terminal distal residues) for STAT5b on mutated GHR is thought to cause the failure of this signal pathway.

Our results suggested significant growth retardation in the −/− pigs have begun since weaning, but no growth failure at the prenatal stage or the neonatal stage. It is the consensus that GH (with a low concentration during gestation) has limited roles in fetal growth, while IGF-I exerts its fundamental effects on prenatal growth both by endocrine and paracrine signaling^40^. In addition, the liver *GHR* mRNA and protein expression before birth and weaning are low in many species, including sheep, pig, rat and mouse^41^,^42^,^43^. Moreover, the immaturity of the GH-IGF-I axis and hypothalamic-pituitary system will also limit the growth-promoting effects of GH in newborns. These data explained why there were no reductions of the fetal or the neonatal growth in our homozygous *GHR* knockout pigs. This result is in accordance with the observation of normal birth size and weight in some postnatal growth defect animals. On the other hand, this result also agrees that transgenic animals with overexpression of GH could not have an evaluated birth size and weight^44^. In contrast, during postnatal growth, GH indirectly stimulates the production of IGF-I in the liver by initialing GH signaling through binding to GHR or directly promoting the growth of target organs. Global deletion of *GHR* would eliminate IGF-I and subsequently deprive the organism of the essential roles of GH in pigs. In particular, liver-specific *GHR* is poorly expressed before 42 days and increases from 42 days onward^42^,^45^. This expression pattern of pig *GHR* correlates with the growth differences observed in our study.

In summary, we generated a miniature pig disease model for human Laron syndrome by knocking out the *GHR* gene. The primary results suggest that these pigs could serve as an ideal disease model for investigating human Laron syndrome by bridging the gap between the mouse model and humans. *GHR* gene knockout pigs could also provide further insight into cancer and life expectancy in patients with *GHR* mutations.

## Methods

### Animal models

All procedures were guaranteed by animal welfare following instructions approved by the China Council on Animal Care and Protocols. All experiments were approved by the Institutional Animal Care and Use Committee of the China Agricultural University (Permit Number: SKLAB-2012–06–01).

### Plasmids and cells

For *in vitro* validation, pig wild-type *GHR* and mutated *GHR* were cloned into the expression vector Psp72-AvITIT-c-flag, obtained as a kind gift from Dr. Xiaojuan Liu (China Agricultural University). This vector used a CAG promoter and SV40 polyA signal to facilitate the expression of foreign genes. Moreover, it contained an EGFP gene that can be used as a marker of transfection efficiency. Spi-luciferase reporter was constructed by cloning 500 bp of sequence upstream of the transcription start site of the rat Spi 2.1 gene. pGL3-Basic vector was purchased from Promega (Promega, USA). Porcine fetal fibroblasts and DF1 cells were used in this work.

### Generation of *GHR* gene knockout pigs

Approximately 1 × 10^6^ fetal fibroblasts were transfected with 2 μg of each ZFNs set 2 left and ZFNs set 2 right vectors (Sigma, USA). After 24 h, the cells were transferred to six 10-cm plates with selective medium containing G418 (500 μg/mL, Promega). The selection process lasted nearly 10 d. Resistant clones were collected using cloning cylinders (Sigma, USA) and transferred to 24-well plates. After 48 h, subconfluent cells were harvested. Half of the cells were subjected to genotyping, and the rest were cryopreserved in liquid nitrogen. After identifying the positive clones, somatic cell nuclear transfer (SCNT) was conducted as described previously using the cryopreserved cells. Each of the surrogate sows received 300 embryos. Pregnancy was determined by abdominal ultrasound examination one month after the SCNT. Approximately 110 d later, piglets were delivered by natural birth.

### Genotyping of *GHR* gene knockout pigs

Genomic DNA was prepared from ear tissues using the phenol method. One pair of *GHR* gene-specific primers was used to identify the genetically modified pigs. The primers are as follows: *GHR*-F: 5′-AAGCGGTGTCTATGTGCTGATTCTC-3′ and *GHR*-R: 5′-ATTGTAATGGGGAGGTTCTGG-3′. PCR reactions were: 95 °C for 5 min; 30 cycles of 95 °C for 30 s, 60 °C for 30 s, then 68 °C for 45 s; and finally held at 68 °C for 7 min. The amplified products were 653 bp in length. The PCR products were cloned into the pMD-19 T vector and were analyzed by Sanger sequencing. PCR products from the targeted genomic region were also subjected to T7E1 cleavage assay; purified PCR products were denatured and annealed in NEB buffer 2 (NEB, USA) using a thermocycler. Hybridized PCR products were digested with T7 endonuclease 1 (NEB, USA) for one hour at 37 °C and then subjected to 2.5% agarose gel electrophoresis or 10% PAGE.

### Quantitative real-time PCR (qRT-PCR)

To detect the transcription levels of *GHR*, *IGF-I*, *JAK2* and *STAT5b*, total RNA was isolated from the ear or liver tissues of the genetically modified pigs using TRIzol (Tiangen, China). The primers used to detect *GHR* mRNA expression were *GHR*-RT-F: 5′-GGATAAAGAGTATGAAGTGCGTGTG-3′ and *GHR*-RT-R: 5′- GATAATTAAGAACCATGGAAACCGG -3′. The primers for *IGF-I* were *IGF*-I-F: 5′-GACGCTCTTCAGTTCGTGTG-3′ and *IGF*-I-R: 5′-TCCTGAACTCCCTCTACTTGTG-3′. The primers for *JAK2* were *JAK2*-F: 5′-GCCAACCTCACCAACATTACA-3′ and *JAK2*-R: 5′-CTGGCTCATCATGCTTGCTG-3′. The primers for *STAT5b* were *STAT5b*-F: 5′-CCAGACCCTGCAGCAGTAC-3′ and *STAT5b*-R: 5′-CCAGATGATCTCCGCCAAC-3′. Pig *GAPDH* was used as the internal control and the primers were: *GAPDH*-RT-F: 5′- ATCACCATCT TCCAGGAGCGA -3′ and *GAPDH*-RT-F: 5′- AGCCTTC TCCATGGTCGTGAA -3′. The expression levels were analyzed using the 2^−ΔΔct^ method.

### Western blotting

Total protein from transfected DF-1 cells or membrane protein from the liver tissues from the transgenic pigs and wild-type pigs was obtained with a protein extraction kit (Beyotime, China). Protein was mixed with loading buffer and then separated on 10% polyacrylamide tris-glycine gels. Separated proteins were electrophoretically transferred to nitrocellulose membranes (Amersham Pharmacia, UK), which were blocked with 5% milk in TBST at 4 °C for one hour. Liver protein was probed with a GHR specific primary antibody (1:500 dilution, Bioss, China). Protein extracted from the transfected DF1 cells was probed with the Flag tag-specific primary antibody (1:2000, Abcam, USA). Then, blots were incubated with goat anti-rabbit secondary antibody (for the GHR primary antibody) or goat anti-mouse secondary antibody (for the Flag tag primary antibody) (1:10,000 dilution, ZSGB-Bio, China) for one hour at room temperature, followed by washes in TBST. The membranes were subjected to luminol-based chemiluminescence with a commercial substrate (Millipore, USA) and Kodak film.

### Enzyme-linked immunosorbent assay (ELISA)

Blood samples were collected from pigs at the age of five months after fasting overnight. The serum was separated by centrifugation at 4 ^°^C for 5 min at 3,600 × rpm and frozen at −80 °C for future assay. The amounts of GH and IGF-I in *GHR* gene-disrupted pigs as well as control pigs were determined using the enzyme-linked immunosorbent assay (ELISA) kit for porcine GH and IGF-I (USCN, China) according to the manufacturer’s instructions. To determine the GH and IGF-I levels in pig serum, we diluted the serum at 1: 5 and 1: 40, respectively. Briefly, 100 μL of the diluted samples were added to the pre-coated wells. After the incubation for two hours at 37 °C, plates were incubated with 100 μL of detection antibody for one hour at 37 °C followed by washing, and then 90 μL HRP solution was added, and subsequently the colorimetric reaction was developed with 50 μL of the substrate TMB. The absorbance of the products was measured at 450 nm using a model 550 microplate reader (Bio-Rad, Hercules, CA).

### Immunofluorescent staining

DF1 cells stably transfected with pig wild-type *GHR* or mutated *GHR* transcript were fixed in 4% PFA. Immunofluorescence was performed as described previously^46^. The Flag tag primary antibody was used at a dilution of 1:500 (Abcam, USA). Cy3-conjugated goat anti-mouse IgG secondary antibody (1:400) was used for the detection of pig GHR (CWBiotech, China). Immunofluorescent staining was observed under an Olympus BX50 Fluorescence Microscope (Olympus, Japan).

### GH binding assay and luciferase assay

The Octet RED96 system (ForteBio, USA) was employed in this assay. The anti-mouse IgG Fc capture chip (ForteBio, USA) was used to bind anti-Flag tag (20 μg/mL) antibody first, and then was bound to the proteins with the Flag tag extracted from the mutated pig GHR or the wild-type pig GHR stably expressing cell clones. The bound proteins then were associated with different amounts of bGH (0 nM, 400 nM, 800 nM, 1600 nM, 3200 nM, 6400 nM) (Prospecbio, USA) and dissociated to obtain kdis and kon. The KD was calculated by kdis/kon. A dual luciferase assay was performed using the dual luciferase assay system (Promega, USA) and a luminometer (Tecan, Switzerland). The results were shown as the mean ± standard deviations (S.D.) from triplicate experiments. Each value was shown as a fold induction normalized to that of the luciferase in cells expressing the wild-type pig GHR under treatment with 50 nM GH and 200 nM Dex, the value of which was set at 1.0.

### Statistical analysis

The experimental data were analyzed by one-way ANOVA and Student’s t test using SPSS15.0 software. The data were expressed as the mean ± standard deviations (S.D.). A *P* value < 0.05 was considered significant.

## Additional Information

**How to cite this article**: Cui, D. *et al*. Generation of a miniature pig disease model for human Laron syndrome. *Sci. Rep*. **5**, 15603; doi: 10.1038/srep15603 (2015).

## Supplementary Material

Supplementary Information

## Figures and Tables

**Table 1:** The optimization of the pig fibroblasts transfected with ZFNs.

ZFNs	ZFNs type	ZFNs amount	mutations
set 1	plasmid	0.5 μg	0/30 (0)
	plasmid	1 μg	2/97 (2.04%)
	plasmid	2 μg	0/32 (0)
	mRNA	2 μg	0/ 95 (0)
	plasmid	4 μg	0/98 (0)
set 2	plasmid	0.5 μg	1/96 (1.04%)
	plasmid	1 μg	3/94 (3.19%)
	plasmid	2 μg	2/66 (3.03%)
	mRNA	2 μg	0/91 (0)
	plasmid	4 μg	18/80 (22.5%)

ZFNs activities were determined by TA cloning and Sanger sequencing.

**Table 2:** The mutation efficiency of ZFNs set 2 in various fibroblasts and at different concentrations.

Fibroblast	ZFNs	Mutateclones	Sequenceclones	mutations
Landrace	set 2 (4 μg)	19	94	19/83 (22.89%)
Landrace	set 2 (6 μg)	13	74	13/74 (17.57%)
Miniature pig	set 2 (6 μg)	14	81	14/81 (17.28%)

The mutation efficiencies of the ZFNs were determined by TA cloning and Sanger sequencing.

**Table 3:** Comparison of the mutation efficiency of ZFNs set 2 in species-specific cell lines.

Species	Target sites	mismatches	mutations
porcine	ACCACCTCCCAATGCAGATGTTCAGAAGGGATGGAT	0	17/96 (17.7%)
bovine	ACCACCaCCCAATaCAGATGTTaAGAtGGGATGGAT	4	0/98 (0)
sheep	ACCACCaCCCAATaCAGATGTTaAGAtGGGATGGAT	4	0/83 (0)
rabbit	ACCACCaCCCAATGCAGATGTTCAGAAGGGATGGAT	1	3/119 (3.4%)
human	AgCACCaCgCAATGCAGATaTTCAGAAgGGATGGAT	5	0/91 (0)

The mutation efficiencies of the ZFNs were determined by TA cloning and Sanger sequencing.

## References

[b1] Laron Z., Pertzelan A. & Mannheimer S. Genetic pituitary dwarfism with high serum concentation of growth hormone—a new inborn error of metabolism? Isr J Med Sci 2, 152–155 (1966). 5916640

[b2] Keret R., Pertzelan A., Zeharia A., Zadik Z. & Laron Z. Growth hormone (hGH) secretion and turnover in three patients with Laron-type dwarfism. Isr J Med Sci 24, 75–79 (1988). 3356536

[b3] Laron Z. . Laron syndrome due to a post-receptor defect: response to IGF-I treatment. Isr J Med Sci 29, 757–763 (1993). 8300382

[b4] Godowski P. J. . Characterization of the human growth hormone receptor gene and demonstration of a partial gene deletion in two patients with Laron-type dwarfism. Proceedings of the National Academy of Sciences of the United States of America 86, 8083–8087 (1989). 2813379 10.1073/pnas.86.20.8083 PMC298219

[b5] Bazan J. F. Structural Design and Molecular Evolution of a Cytokine Receptor Superfamily. Proceedings of the National Academy of Sciences of the United States of America 87, 6934–6938, doi: 10.1073/Pnas.87.18.6934 (1990). 2169613 PMC54656

[b6] Harding P. A. . *In vitro* mutagenesis of growth hormone receptor Asn-linked glycosylation sites. Molecular and cellular endocrinology 106, 171–180 (1994). 7895905 10.1016/0303-7207(94)90200-3

[b7] Baumann G., Stolar M. W., Amburn K., Barsano C. P. & DeVries B. C. A specific growth hormone-binding protein in human plasma: initial characterization. The Journal of clinical endocrinology and metabolism 62, 134–141, doi: 10.1210/jcem-62-1-134 (1986). 3940261

[b8] Argetsinger L. S. . Identification of Jak2 as a Growth-Hormone Receptor-Associated Tyrosine Kinase. Cell 74, 237–244, doi: 10.1016/0092-8674(93)90415-M (1993). 8343952

[b9] Gronowski A. M. & Rotwein P. Rapid changes in nuclear protein tyrosine phosphorylation after growth hormone treatment *in vivo*. Identification of phosphorylated mitogen-activated protein kinase and STAT91. The Journal of biological chemistry 269, 7874–7878 (1994). 7510676

[b10] Moller C., Hansson A., Enberg B., Lobie P. E. & Norstedt G. Growth hormone (GH) induction of tyrosine phosphorylation and activation of mitogen-activated protein kinases in cells transfected with rat GH receptor cDNA. The Journal of biological chemistry 267, 23403–23408 (1992). 1385420

[b11] Wood T. J., Haldosen L. A., Sliva D., Sundsthom M. & Norstedt G. Stimulation of kinase cascades by growth hormone: a paradigm for cytokine signaling. Progress in nucleic acid research and molecular biology 57, 73–94 (1997). 9175431 10.1016/s0079-6603(08)60278-0

[b12] Boisclair Y. R., Rhoads R. P., Ueki I., Wang J. & Ooi G. T. The acid-labile subunit (ALS) of the 150 kDa IGF-binding protein complex: an important but forgotten component of the circulating IGF system. The Journal of endocrinology 170, 63–70 (2001). 11431138 10.1677/joe.0.1700063

[b13] Laron Z. Laron syndrome (primary growth hormone resistance or insensitivity): the personal experience 1958–2003. The Journal of clinical endocrinology and metabolism 89, 1031–1044, doi: 10.1210/jc.2003-031033 (2004). 15001582

[b14] Fintini D., Brufani C. & Cappa M. Profile of mecasermin for the long-term treatment of growth failure in children and adolescents with severe primary IGF-1 deficiency. Therapeutics and clinical risk management 5, 553–559 (2009). 19707272 10.2147/tcrm.s6178 PMC2724186

[b15] Zhou Y. . A mammalian model for Laron syndrome produced by targeted disruption of the mouse growth hormone receptor/binding protein gene (the Laron mouse). Proceedings of the National Academy of Sciences of the United States of America 94, 13215–13220 (1997). 9371826 10.1073/pnas.94.24.13215 PMC24289

[b16] Agarwal S. K., Cogburn L. A. & Burnside J. Dysfunctional growth hormone receptor in a strain of sex-linked dwarf chicken: evidence for a mutation in the intracellular domain. The Journal of endocrinology 142, 427–434 (1994). 7964293 10.1677/joe.0.1420427

[b17] List E. O. . Endocrine parameters and phenotypes of the growth hormone receptor gene disrupted (GHR−/−) mouse. Endocrine reviews 32, 356–386, doi: 10.1210/er.2010-0009 (2011). 21123740 PMC3365798

[b18] Laron Z. & Kopchick J. J. Laron syndrom- from mouse to man 124–135 (Springer-Verlag Berlin Heidelberg, 2011).

[b19] Isaksson O. G., Lindahl A., Nilsson A. & Isgaard J. Mechanism of the stimulatory effect of growth hormone on longitudinal bone growth. Endocrine reviews 8, 426–438, doi: 10.1210/edrv-8-4-426 (1987). 3319530

[b20] Dai Y. . Targeted disruption of the alpha1,3-galactosyltransferase gene in cloned pigs. Nature biotechnology 20, 251–255, doi: 10.1038/nbt0302-251 (2002). 11875425

[b21] Lai L. . Production of alpha-1,3-galactosyltransferase knockout pigs by nuclear transfer cloning. Science 295, 1089–1092, doi: 10.1126/science.1068228 (2002). 11778012

[b22] Rogers C. S. . Production of CFTR-null and CFTR-DeltaF508 heterozygous pigs by adeno-associated virus-mediated gene targeting and somatic cell nuclear transfer. The Journal of clinical investigation 118, 1571–1577, doi: 10.1172/JCI34773 (2008). 18324337 PMC2265103

[b23] Matsunari H. & Nagashima H. Application of genetically modified and cloned pigs in translational research. The Journal of reproduction and development 55, 225–230 (2009). 19571468 10.1262/jrd.20164

[b24] Horak V., Fortyn K., Hruban V. & Klaudy J. Hereditary melanoblastoma in miniature pigs and its successful therapy by devitalization technique. Cellular and molecular biology 45, 1119–1129 (1999). 10644016

[b25] Carlson D. F., Fahrenkrug S. C. & Hackett P. B. Targeting DNA With Fingers and TALENs. Molecular therapy. Nucleic acids 1, e3, doi: 10.1038/mtna.2011.5 (2012). 23344620 PMC3381595

[b26] Carlson D. F. . Efficient TALEN-mediated gene knockout in livestock. Proceedings of the National Academy of Sciences of the United States of America 109, 17382–17387, doi: 10.1073/pnas.1211446109 (2012). 23027955 PMC3491456

[b27] Chung C. S., Etherton T. D. & Wiggins J. P. Stimulation of swine growth by porcine growth hormone. Journal of animal science 60, 118–130 (1985). 3882649 10.2527/jas1985.601118x

[b28] Jorgensen K. D., Kledal T. S. A., Svendsen O. & Skakkeboek N. E. The Gottingen minipig as a model for studying effects on male fertility. Scand J Lab Anim Sci 25, 161–169 (1998).

[b29] Honig J. F. & Merten H. A. Subperiosteal versus epiperiosteal forehead augmentation with hydroxylapatite for aesthetic facial contouring: experimental animal investigation and clinical application. Aesthetic plastic surgery 17, 93–98 (1993). 8390778 10.1007/BF02274727

[b30] Martiniakova M., Grosskopf B., Omelka R., Vondrakova M. & Bauerova M. Differences among species in compact bone tissue microstructure of mammalian skeleton: Use of a discriminant function analysis for species identification. J Forensic Sci 51, 1235–1239, doi: Doi 10.1111/J.1556-4029.2006.00260.X (2006). 17199608

[b31] Miller E. R. & Ullrey D. E. The Pig as a Model for Human-Nutrition. Annu Rev Nutr 7, 361–382, doi: Doi 10.1146/Annurev.Nu.07.070187.002045 (1987). 3300739

[b32] Bentouimou N., Vaugelade P., Berard F., Cherbut C. & DarcyVrillon B. Compared metabolic effects of seaweed fibres in pigs and humans 57–60 (Eaap Public, 1997).

[b33] Litten-Brown J. C., Corson A. M. & Clarke L. Porcine models for the metabolic syndrome, digestive and bone disorders: a general overview. Animal : an international journal of animal bioscience 4, 899–920, doi: 10.1017/S1751731110000200 (2010). 22444262

[b34] Swindle M. M. & Smith A. C. Comparative anatomy and physiology of the pig. Scand J Lab Anim Sci 25, 11–21 (1998).

[b35] David A. . Evidence for a continuum of genetic, phenotypic, and biochemical abnormalities in children with growth hormone insensitivity. Endocrine reviews 32, 472–497, doi: 10.1210/er.2010-0023 (2011). 21525302

[b36] Kaabi Y. Growth hormone and its receptor: A molecular insight. Saudi Journal for Health Sciences 1, 61, doi: 10.4103/2278-0521.100942 (2012).

[b37] Wood T. J. J. . Mediation of Growth Hormone-Dependent Transcriptional Activation by Mammary-Gland Factor Stat-5. Journal of Biological Chemistry 270, 9448–9453 (1995). 7721871 10.1074/jbc.270.16.9448

[b38] Iida K. . Functional characterization of truncated growth hormone (GH) receptor-(1-277) causing partial GH insensitivity syndrome with high GH-binding protein. J Clin Endocr Metab 84, 1011–1016, doi: 10.1210/Jc.84.3.1011 (1999). 10084588

[b39] Tiulpakov A. . A novel C-terminal growth hormone receptor (GHR) mutation results in impaired GHR-STAT5 but normal STAT-3 signaling. J Clin Endocr Metab 90, 542–547, doi: 10.1210/Jc.2003-2133 (2005). 15536163

[b40] Gluckman P. D. & Pinal C. S. Regulation of fetal growth by the somatotrophic axis. The Journal of nutrition 133, 1741S–1746S (2003). 12730493 10.1093/jn/133.5.1741S

[b41] Baumbach W. R. & Bingham B. One class of growth hormone (GH) receptor and binding protein messenger ribonucleic acid in rat liver, GHR1, is sexually dimorphic and regulated by GH. Endocrinology 136, 749–760, doi: 10.1210/endo.136.2.7835307 (1995). 7835307

[b42] Liu J., Carroll J. A., Matteri R. L. & Lucy M. C. Expression of two variants of growth hormone receptor messenger ribonucleic acid in porcine liver. Journal of animal science 78, 306–317 (2000). 10709921 10.2527/2000.782306x

[b43] Pratt S. L. & Anthony R. V. The growth hormone receptor messenger ribonucleic acid present in ovine fetal liver is a variant form. Endocrinology 136, 2150–2155, doi: 10.1210/endo.136.5.7720664 (1995). 7720664

[b44] Palmiter R. D., Norstedt G., Gelinas R. E., Hammer R. E. & Brinster R. L. Metallothionein-human GH fusion genes stimulate growth of mice. Science 222, 809–814 (1983). 6356363 10.1126/science.6356363

[b45] Breier B. H., Gluckman P. D., Blair H. T. & McCutcheon S. N. Somatotrophic receptors in hepatic tissue of the developing male pig. The Journal of endocrinology 123, 25–31 (1989). 2809488 10.1677/joe.0.1230025

[b46] Yu Z. . The Grainyhead-like epithelial transactivator Get-1/Grhl3 regulates epidermal terminal differentiation and interacts functionally with LMO4. Developmental biology 299, 122–136, doi: 10.1016/j.ydbio.2006.07.015 (2006). 16949565

