# Supplementary material for: Generation of a miniature pig disease model for human Laron syndrome
Source: Sci Rep. 2015 Oct 29;5:15603. doi: 10.1038/srep15603 (PMC4625145; doi:10.1038/srep15603)
Supplement: Supplementary Information [file srep15603-s1.pdf]

Generation of a miniature pig disease model for human Laron syndrome

Dan Cui, Fang Li, Qiuyan Li, Jia Li, Yaofeng Zhao, Xiaoxiang Hu, Ran Zhang*, Ning Li*

State Key Laboratory for Agrobiotechnology, China Agricultural University, Beijing 100193, China

Corresponding author: Ning Li and Ran Zhang

State Key Laboratory for Agrobiotechnology, China Agricultural University, Beijing 100193, China

Tel: +86 10 6273 3323; Fax: +86 10 6273 3904

*E-mail: ninglcau@cau.edu.cn; zhangran0628@cau.edu.cn

Supplementary Materials

Supplementary Figure S1. Identification of F0 heterozygous pigs

Supplementary Figure S2. Identification of F1 $GHR^{+/4bp}$ pigs by T7E1 cleavage

Supplementary Figure S3. Identification of *GHR* mRNA expression in the liver tissues of F2 pigs

Supplementary Figure S4. GHBP levels in the F2 generation

Supplementary Figure S5. Reduced bone growth in -/- pigs

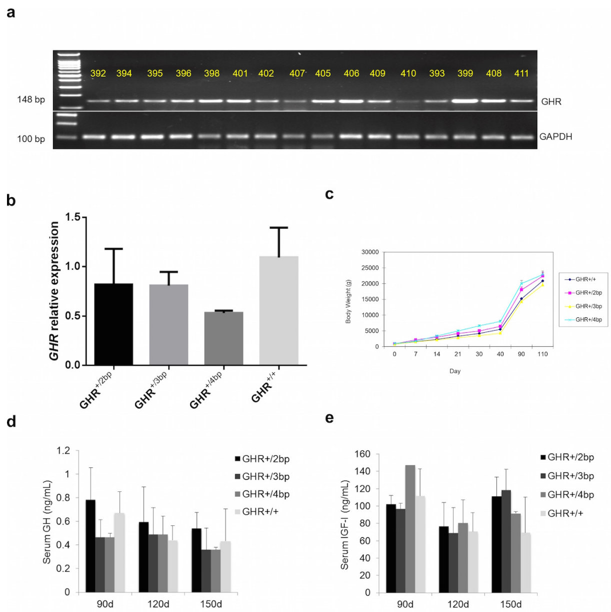

Supplementary Figure S1 | Identification of F0 heterozygous pigs. Detection and quantification of *GHR* mRNA expression in F0 pigs by RT-PCR (a) and qRT-PCR (b). Numbers indicate individual F0 pigs; GHR$^{+/4bp}$, GHR$^{+/2bp}$, GHR$^{+/3bp}$ are the different genotypes in the F0 heterozygous pigs. The expression levels were determined by the expression relative to *GAPDH* (an internal control). The data were combined from three independent experiments; the bars represent the means ± SD. (c) No significant difference in body weight was seen between F0 heterozygous pigs and wild-type controls. Neither a significantly increased level of GH (d) nor a significantly reduced level of IGF-I (e) was observed in F0 heterozygous pigs compared with the wild-type controls. The bars represent the means ± SD.

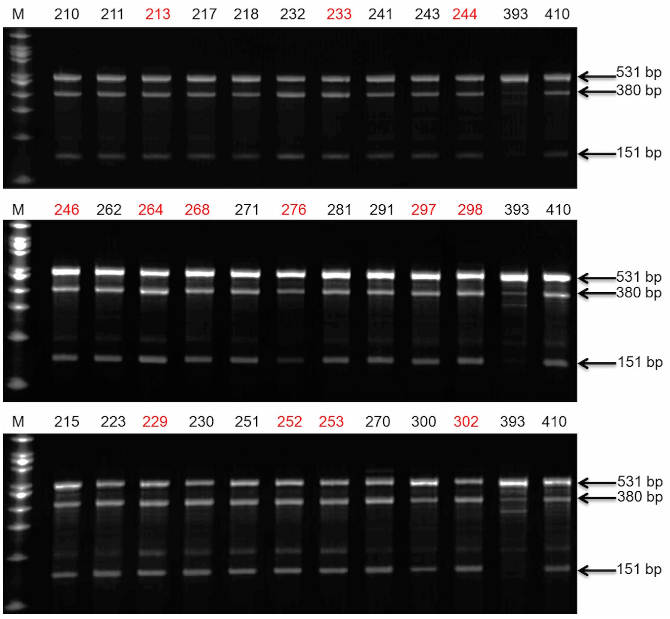

Supplementary Figure S2 | Identification of F1 GHR$^{+/4bp}$ pigs by T7E1 cleavage. The products after cleavage were separated on 2.5% agarose; the expected sizes are 380 bp and 151 bp after T7E1 cleavage; all the numbers represent the positive GHR$^{+/4bp}$ pigs in the F1 generation; red numbers indicate the female GHR$^{+/4bp}$ pigs.

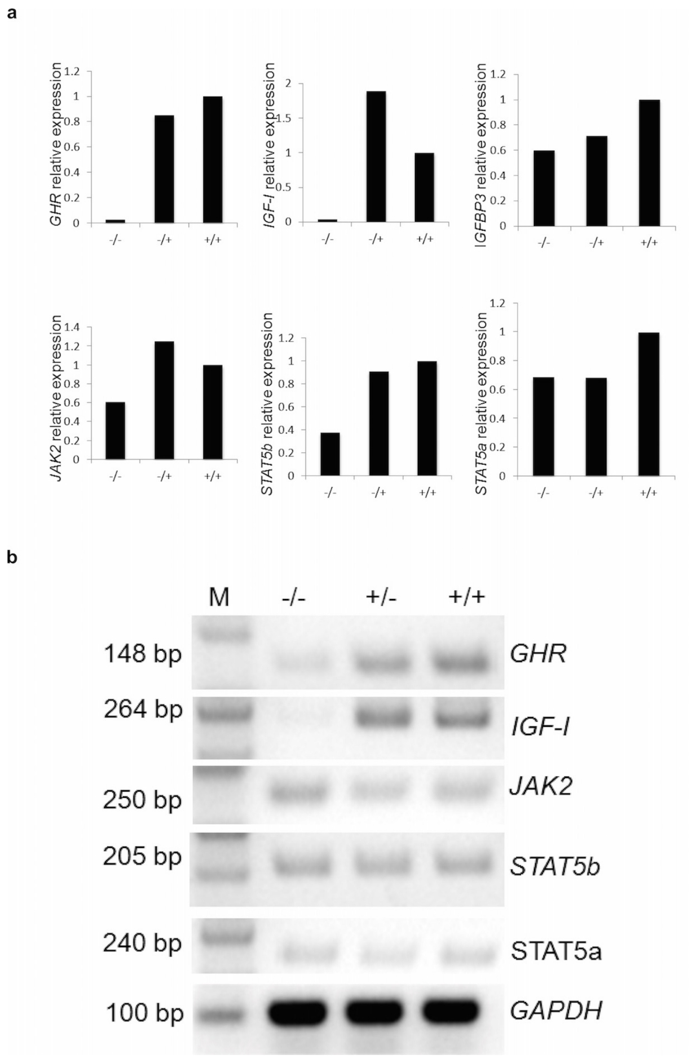

Supplementary Figure S3 | Identification of *GHR* mRNA expression in the liver tissues of F2 pigs. (a) Quantification of *GHR*, *IGF-I*, *IGFBP3*, *JAK2*, *STAT5b* and *STAT5a* mRNA expression in the liver tissues of F2 pigs by qRT-PCR. The expression levels were determined by the expression relative to *GAPDH* (an internal control); the data were combined from three independent experiments, with one pig

per group. (b) RT-PCR results of the liver expression of *GHR*, *IGF-I*, *IGFBP3*, *JAK2*, *STAT5b* and *STAT5a.*

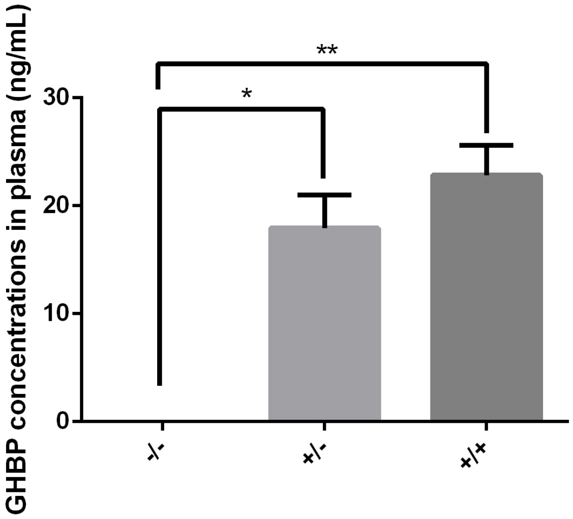

Supplementary Figure S4 | GHBP levels in the F2 generation. The five-month-old -/- pigs showed an absence of GHBP in plasma. The -/-, +/- ($GHR^{+/4bp}$) and +/+ ($GHR^{+/+}$) represent different genotypes in the F2 generation; the bars represent the means ± SD (at least three pigs per group); ***P*<0.01, **P*<0.05.

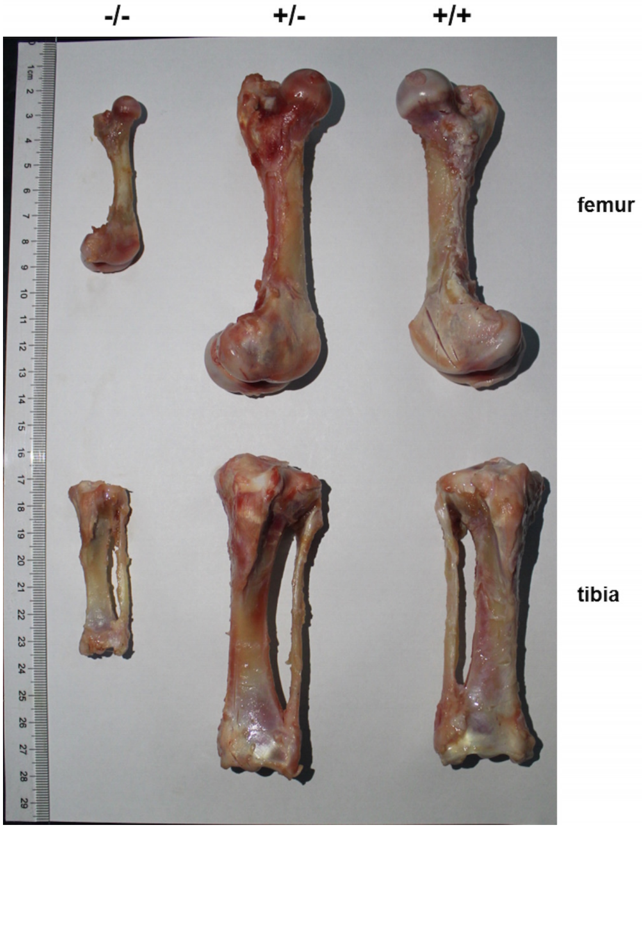

Supplementary Figure S5 | Reduced bone growth in the -/- pigs. The femur and tibia from three-and-half-month-old pigs are shown; the -/-, +/- ($GHR^{+/4bp}$) and +/+ ($GHR^{+/+}$) represent different genotypes in the F2 generation; with one pig per group.
